# Efficient production of mycosporine-like amino acids, natural sunscreens, in *Yarrowia lipolytica*

**DOI:** 10.1186/s13068-023-02415-y

**Published:** 2023-10-29

**Authors:** Hyunbin Jin, Sojeong Kim, Daeyeol Lee, Rodrigo Ledesma-Amaro, Ji-Sook Hahn

**Affiliations:** 1 School of Chemical and Biological Engineering, Institute of Chemical Processes, Seoul National University, 1 Gwanak-ro, Gwanak-gu, Seoul, 08826 Republic of Korea; 2 Department of Bioengineering and Imperial College Centre for Synthetic Biology, Imperial College London, London, SW7 2AZ UK

**Keywords:** Metabolic engineering, Mycosporine-like amino acids, Porphyra-334, Shinorine, Sunscreen, *Yarrowia lipolytica*

## Abstract

**Background:**

Mycosporine-like amino acids (MAAs), including shinorine and porphyra-334, are gaining attention as safe natural sunscreens. The production of MAAs has been achieved in diverse microbial hosts, including *Saccharomyces cerevisiae*. While *S. cerevisiae* is the most extensively studied model yeast, the oleaginous yeast *Yarrowia lipolytica* has emerged as a promising candidate for the synthesis of valuable products. In this study, we explored the potential of *Y. lipolytica* as a host for producing MAAs, utilizing its advantages such as a robust pentose phosphate pathway flux and versatile carbon source utilization.

**Results:**

We produced MAAs in *Y. lipolytica* by introducing the MAA biosynthetic genes from cyanobacteria *Nostoc punctiforme* and *Anabaena variabilis.* These genes include *mysA*, *mysB*, and *mysC* responsible for producing mycosporine-glycine (MG) from sedoheptulose 7-phosphate (S7P). The two strains utilize different enzymes, D-Ala-D-Ala ligase homologue (MysD) in *N. punctiforme* and NRPS-like enzyme (MysE) in *A. variabilis*, for amino acid conjugation to MG. MysE specifically generated shinorine, a serine conjugate of MG, while MysD exhibited substrate promiscuity, yielding both shinorine and a small amount of porphyra-334, a threonine conjugate of MG. We enhanced MAAs production by selecting *mysA*, *mysB*, and *mysC* from *A. variabilis* and *mysD* from *N. punctiforme* based on their activities. We further improved production by strengthening promoters, increasing gene copies, and introducing the xylose utilization pathway. Co-utilization of xylose with glucose or glycerol increased MAAs production by boosting the S7P pool through the pentose phosphate pathway. Overexpressing *GND1* and *ZWF1*, key genes in the pentose phosphate pathway, further enhanced MAAs production. The highest achieved MAAs level was 249.0 mg/L (207.4 mg/L shinorine and 41.6 mg/L of porphyra-334) in YP medium containing 10 g/L glucose and 10 g/L xylose.

**Conclusions:**

*Y. lipolytica* was successfully engineered to produce MAAs, primarily shinorine. This achievement involved the introduction of MAA biosynthetic genes from cyanobacteria, establishing xylose utilizing pathway, and overexpressing the pentose phosphate pathway genes. These results highlight the potential of *Y. lipolytica* as a promising yeast chassis strain for MAAs production, notably attributed to its proficient expression of MysE enzyme, which remains non-functional in *S. cerevisiae*, and versatile utilization of carbon sources like glycerol.

**Supplementary Information:**

The online version contains supplementary material available at 10.1186/s13068-023-02415-y.

## Background

Sunscreen is widely used to protect skin from damage caused by ultraviolet (UV) radiation. However, the organic and inorganic materials used in current sunscreens have raised concerns regarding their impact on health and the environment [1]. As a result, there is a growing demand for natural sunscreens that are safe for both humans and the planet. Mycosporine-like amino acids (MAAs) are UV-absorbing materials produced in various organisms, especially in marine organisms such as algae and cyanobacteria exposed to strong sunlight [2, 3]. To date, more than 30 MAAs have been identified, which have either mono-substituted cyclohexanone or di-substituted cyclohexenimine structures [2, 4]. Their absorption spectra range from 310 to 360 nm, covering both UV-A (315–400 nm) and UV-B (280–315 nm) wavelengths that reach the Earth's surface [4, 5]. In addition to their ability to absorb UV radiation, MAAs have been found to possess anti-inflammatory and antioxidant properties, making them appealing ingredients for use in cosmetics and pharmaceuticals [6, 7].

MAAs can be synthesized from sedoheptulose 7-phosphate (S7P), an intermediate in the pentose phosphate pathway, or from 3-dehydroquinate, an intermediate of shikimate pathway [8]. The biosynthetic pathway producing MAAs from S7P involves the production of 4-deoxygadusol (4-DG) through serial actions of demethyl-4-deoxygadusol synthase (DDGS) and O-methyltransferase (O-MT). Then ATP-grasp enzyme conjugates glycine to 4-DG, producing mycosporine-glycine (MG) [9]. The conjugation of additional amino acids to MG is catalyzed by one of the two distinct types of enzymes: non-ribosomal peptide synthetase (NRPS)-like enzyme and D-Ala-D-Ala ligase homologue. This process results in di-substituted MAAs, such as shinorine and porphyra-334, which are formed by the conjugation of serine and threonine to MG, respectively [9]. The MAA biosynthetic genes are present in gene clusters together with genes encoding transporters and other modifying enzymes in prokaryotes. The genes are often named *mysA* (DDGS), *mysB* (O-MT), *mysC* (ATP-grasp enzyme), *mysD* (D-Ala-D-Ala ligase homologue), and *mysE* (NRPS-like enzyme). Although MAAs extracted from red algae are commercially available, the low yield and high cost of extraction make it impractical for large-scale production. Therefore, producing MAAs in heterologous microbial hosts are seen as a promising alternative [10–14].

Previously, we successfully engineered *Saccharomyces cerevisiae* to produce MAAs by introducing the MAA biosynthetic genes from cyanobacteria [14–16]. To enhance shinorine production, increasing the pool of S7P, the precursor for MAA biosynthesis, was critical. We accomplished this using xylose as a co-substrate with glucose and reducing the glycolytic flux, thereby diverting the carbon flux towards the pentose phosphate pathway. While *S. cerevisiae* is the most extensively studied model yeast with desirable properties for industrial applications, *Yarrowia lipolytica* has emerged as a promising host to produce various value-added products [17]. *Y. lipolytica* is particularly well-suited for producing oleochemicals due to its high lipid production capacity. Compared to *S. cerevisiae*, *Y. lipolytica* has a higher flux towards the pentose phosphate pathway to supply NADPH, which is essential for lipid synthesis [18]. Moreover, *Y. lipolytica* exhibits versatility in carbon source consumption, encompassing glycerol, alkane, and lipid, in addition to glucose [19]. With its Generally Recognized as Safe (GRAS) status, *Y. lipolytica* finds utility in various sectors, including pharmaceuticals, cosmetics, and the food industry. In this study, we took advantage of *Y. lipolytica*'s high pentose phosphate pathway flux to investigate its potential as a host for producing MAAs. Compared to *S. cerevisiae*, *Y. lipolytica* exhibited distinct characteristics and advantages as a host for MAAs production.

## Results

### Introduction of MAA biosynthetic pathway into *Y. lipolytica*

**Fig. 1:**
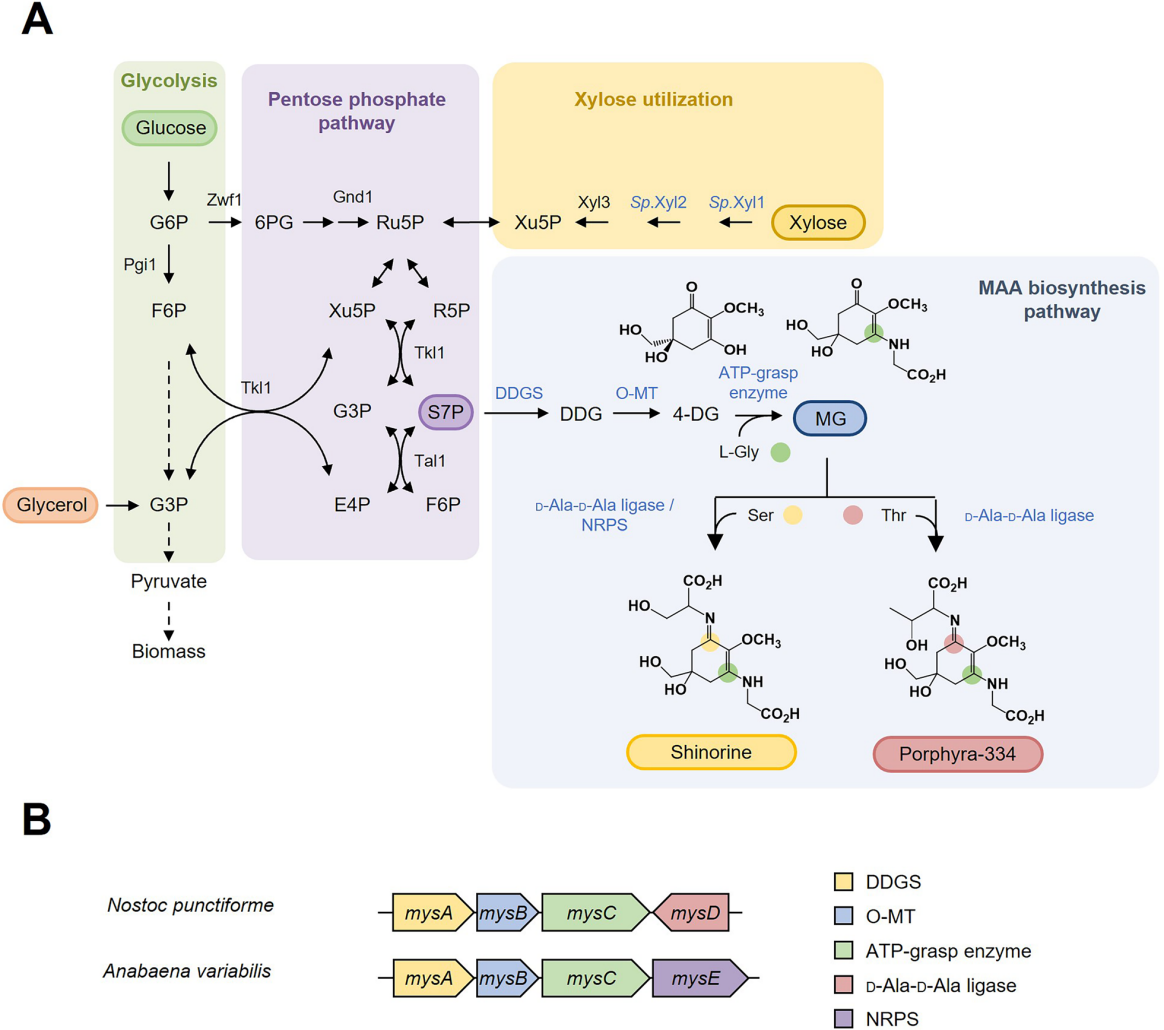
Biosynthesis of MAAs **A** Metabolic pathways for the production of shinorine and porphyra-334 in *Y. lipolytica*. MAAs are produced from S7P via four enzymatic reactions. Heterologous genes were indicated as blue. **B** MAA biosynthetic gene clusters of *A. variabilis* and *N. punctiforme* used in this study. *G6P* glucose 6-phosphate, *F6P* fructose 6-phosphate, *G3P* glyceraldehyde 3-phosphate, *6PG* 6-phosphogluconate, *Ru5P* ribulose 5-phosphate, *R5P* ribose 5-phosphate, *Xu5P* xylulose 5-phosphate, *S7P* sedoheptulose 7-phosphate, *E4P* erythrose 4-phosphate, *DDG* demethyl-4-deoxygadusol, *4-DG* 4-deoxygadusol, *MG* mycosporine-glycine, *DDGS* DDG synthase, *O-MT* O-methyltransferase

**Fig. 2:**
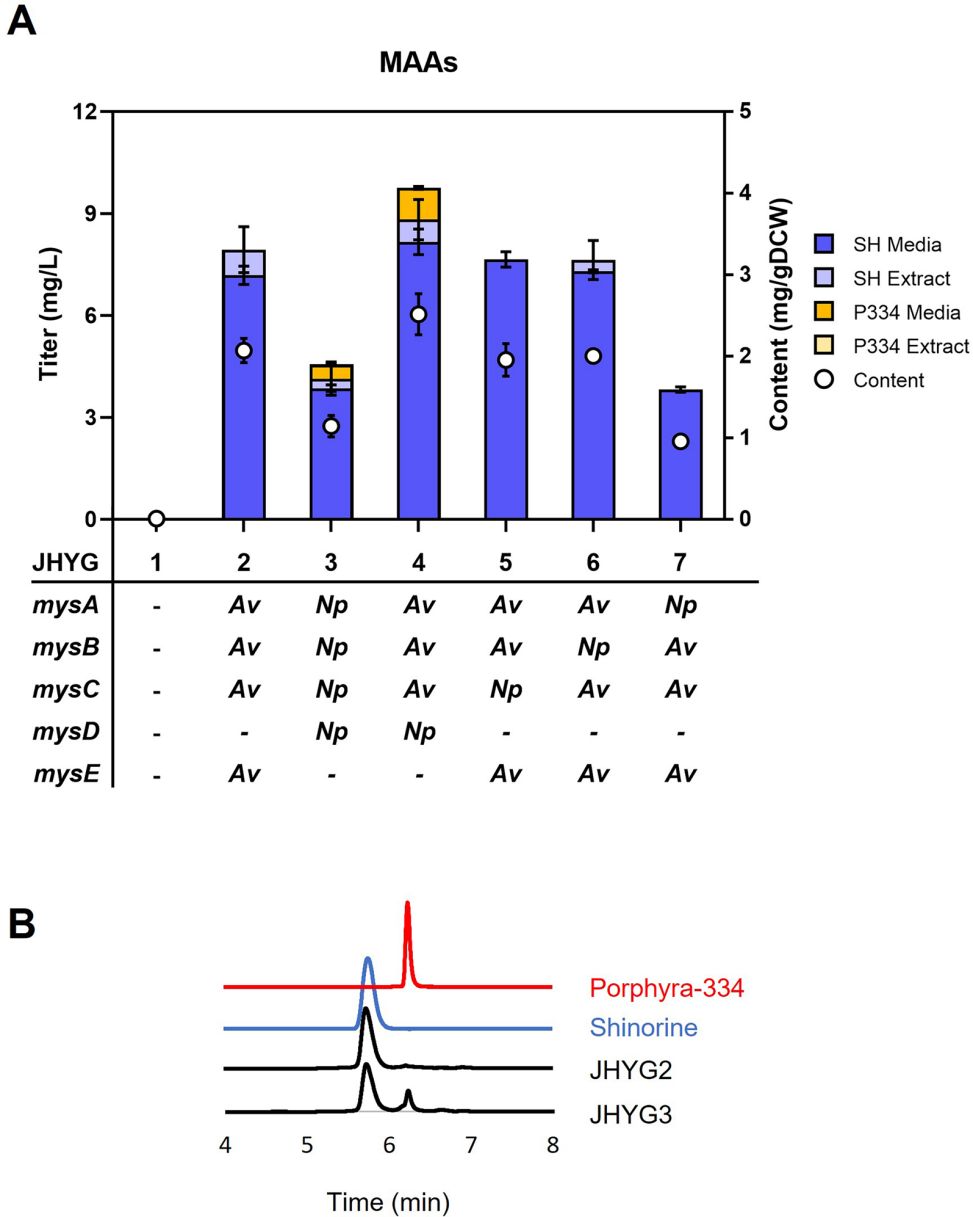
Production of MAAs in *Y. lipolytica* through the introduction of MAA biosynthetic genes. **A** The JHYG 1–7 strains containing the indicated MAA biosynthetic genes were cultured in SC media containing 20 g/L of glucose. The level of MAAs in media and cell extracts was measured after 120 h of cultivation. Each measurement represents the mean ± standard deviation of triplicate samples. **B** HPLC spectra of culture supernatants of JHYG1 and JHYG2 along with the standards for shinorine and porphyra-334. *SH* shinorine; *P334* porphyra-334; *media* extracellular concentration; *extract* intracellular concentration; *Av* *A. variabilis*, *Np* *N. punctiforme*

To produce MAAs in *Y. lipolytica*, we selected two MAA biosynthetic gene clusters from *Nostoc punctiforme* and *Anabaena variabilis*. Both gene clusters contain genes encoding DDGS (*mysA*), O-MT (*mysB*), and ATP-grasp enzyme (*mysC*), which are involved in the production of MG from S7P (Fig. 1A, B). For the additional conjugation of an amino acid to MG, the two strains use different enzymes: D-Ala-D-Ala ligase homologue (MysD) in *N. punctiforme* and NRPS-like enzyme (MysE) in *A. variabilis* (Fig. 1A, B). MysD enzymes have been found to exhibit substrate promiscuity, conjugating various amino acids in addition to their preferred amino acids [20, 21]. On the other hand, MysE of *A. variabilis* specifically produced shinorine [9]. Each gene sequence, optimized for *Y. lipolytica*, was integrated into the genome under the control of a strong P_*TEF(406)*_ promoter and T_*CYC1*_ terminator. JHYG2 and JHYG3 strains expressing the MAA biosynthetic gene cluster from *A. variabilis* and *N. punctiforme*, respectively, were grown in minimal SC media and the production of MAAs was detected. Both strains produced shinorine, with a serine conjugation to MG, as the major product, and the produced MAAs were mostly secreted to the media (Fig. 2A). JHYG2 and JHYG3 produced 7.9 mg/L and 4.1 mg/L of total shinorine, respectively. Consistent with the substrate promiscuity of MysD, JHYG3 strain also showed a minor production of porphyra-334 (0.4 mg/L), which is synthesized through the conjugation of threonine to MG (Fig. 2A, B). On the contrary, JHYG2 exclusively produced shinorine (Fig. 2A, B), supporting the high specificity of MysE towards serine. JHYG2 produced higher level of total MAAs (7.9 mg/L) than JHYG3 (4.6 mg/L).

To identify the optimum set of biosynthetic genes, we generated strains where each gene in the *A. variabilis* biosynthetic gene cluster was replaced with the corresponding gene from *N. punctiforme*, one at a time. JHYG4 expressing *Np.mycD* together with *mysA*, *B*,* C* genes from *A. variabilis* produced higher level of total MAAs compared with JHYG2, suggesting the higher activity of *Np.*MysD than *Av.*MysE (Fig. 2A). The substitution of *Av.mysB* or *Av.mysC* with their corresponding genes from *N. punctiforme* (JHYG5 and JHYG6) did not significantly impact the production level of MAAs (Fig. 2A). This observation implies that *mysB* and *mysC* genes from both *N. punctiforme* and *A. variabilis* exhibit comparable activities. On the other hand, substituting *Av.mysA* with *Np.mysA* (JHYG7) led to a significant reduction in MAAs production, similar to that observed in JHYG3 (Fig. 2A). These findings suggest that *Av*.MysA possesses a higher activity than *Np*.MysA, which is consistent with our previous finding when these genes were expressed in *S. cerevisiae* [16]. Based on these results, we selected *Av.mysA, B, C* and *Np.mysD* genes to further improve MAAs production.

**Fig. 3:**
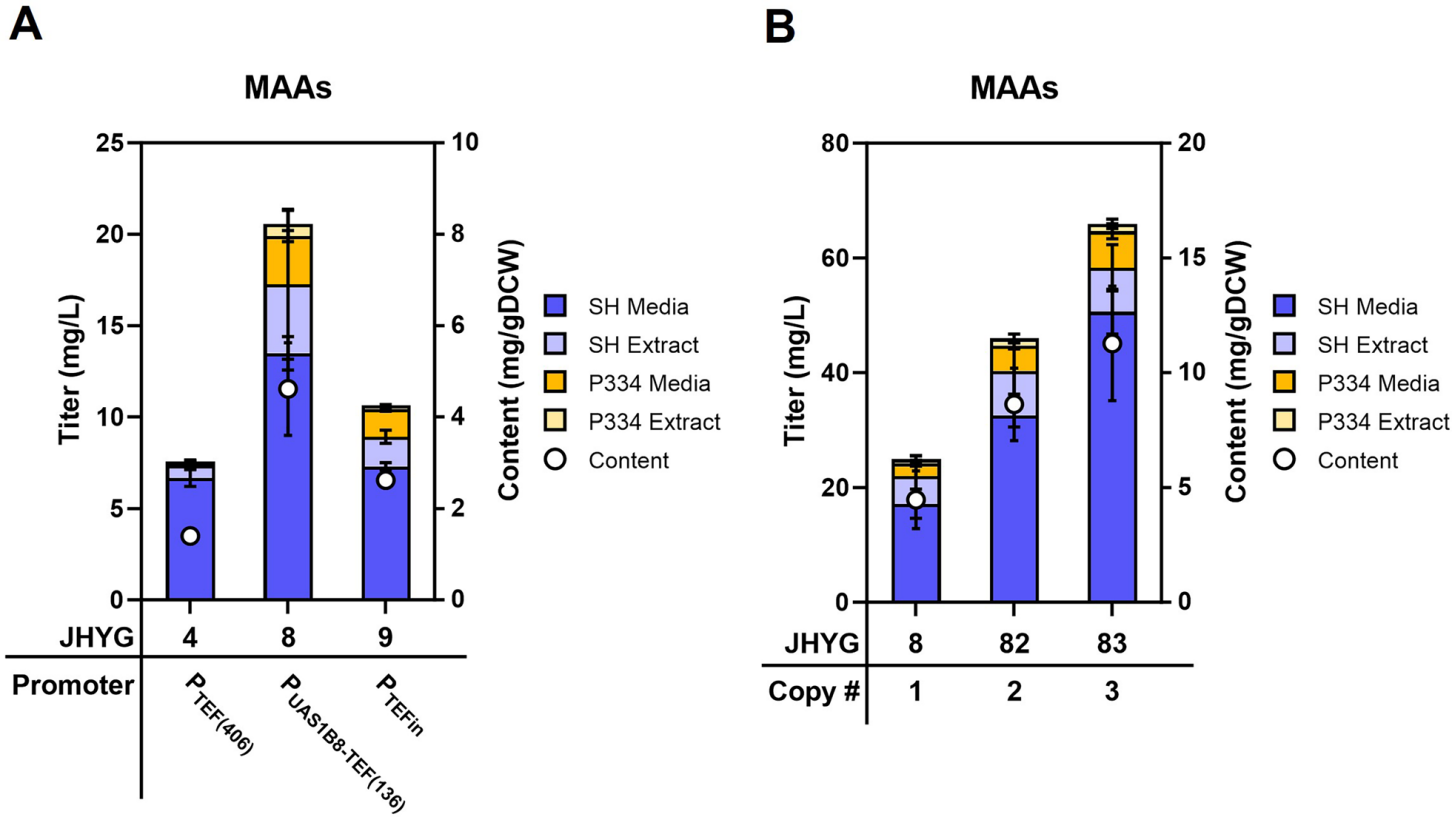
**A** Enhancement of MAAs production through promoter replacement. **B** Increase in MAAs production by the copy number of genes involved in MAA biosynthesis. Cells were grown in SC media containing 20 g/L of glucose for 120 h to measure MAAs levels. Each value represents the mean ± standard deviation of triplicate samples

Subsequently, we compared various promoters for expressing the MAA biosynthetic genes. In addition to the P_*TEF(406)*_ promoter, we tested two other strong promoters, P_*UAS1B8-TEF(136)*_ and P_*TEFin*_, for gene expression (22, 23). Among the three strains, JHYG8, expressing the four biosynthetic genes using the P_*UAS1B8-TEF(136)*_ promoter, showed the highest production levels of MAAs (Fig. 3A). To further enhance the production of MAAs, up to three copies of the P_*UAS1B8-TEF(136)*_-controlled gene expression cassettes were integrated into genome. The MAAs production levels showed a positive correlation with the gene copy number (Fig. 3B). The JHYG83 strain, which carried three copies of each biosynthetic gene, produced 58.3 mg/L of shinorine and 7.6 mg/L of porphyra-334, achieving about 2.6-fold increase in total MAAs level compared with JHYG8 harboring one copy of the biosynthetic gene set (Fig. 3B).

### Impact of carbon sources and media on MAAs production

**Fig. 4:**
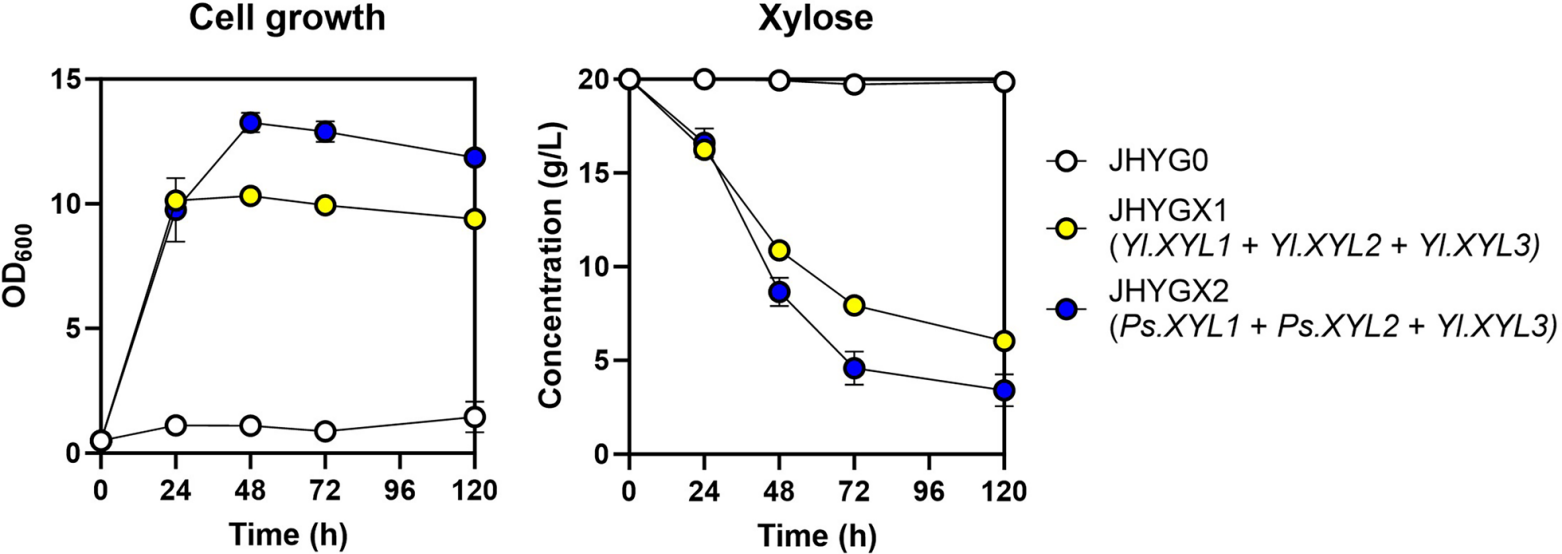
Introduction of xylose utilization pathway in *Y. lipolytica*. JHYGX1 and JHYGX2 Strains expressing the xylose consumption genes *XYL*1, *XYL2*, *XYL3* from *Y. lipolytica* (*Yl*) or *XYL1* and *XYL2* from *P. stipitis* (*Ps*), and *XYL3* from *Y. lipolytica*, respectively, were grown in SC media containing 20 g/L xylose. Cell growth and xylose consumption were detected. Each value represents the mean ± standard deviation of triplicate samples

*Y. lipolytica* has the ability to utilize various alternative carbon sources, including glycerol and oils. Despite possessing genes for xylose utilization, the prevailing evidence indicates that *Y. lipolytica* cannot naturally consume xylose unless genetic manipulation or adaptive evolution techniques are applied [24–27]. Accordingly, the parental strain JHYG0 could hardly grow on xylose (Fig. 4). The xylose utilization pathway, mediated by xylose reductase (XR), xylitol dehydrogenase (XDH), and xylulose kinase (XK), converts xylose into xylulose 5-phosphate, which then enters the pentose phosphate pathway. As S7P is a precursor for MAA biosynthesis and an intermediate of the pentose phosphate pathway, utilizing xylose as a carbon source has been proven to be an effective method for increasing MAAs production in *S. cerevisiae* [15].

To enhance xylose utilization, we overexpressed endogenous xylose-utilizing genes *XYL1*, *XYL2*, and *XYL3* encoding XR, XDH, and XK, respectively, controlled by strong promoter P_*TEF(406)*_ in JHYG0. We also tested overexpression of *XYL1* and *XYL2* genes from *Pichia stipitis* along with the endogenous *XYL3* gene [25]. Overexpression of the xylose-utilizing genes enabled growth on xylose as a sole carbon source. Furthermore, *XYL1* and *XYL2* from *P. stipitis* exhibited greater efficacy compared to the endogenous genes in xylose consumption (Fig. 4). Based on these findings, we introduced the P_*TEF(406)*_-controlled *Ps.XYL1*, *Ps.XYL2*, and *Yl.XYL3* into the JHYG83 strain, generating JHYG831 strain.

**Fig. 5:**
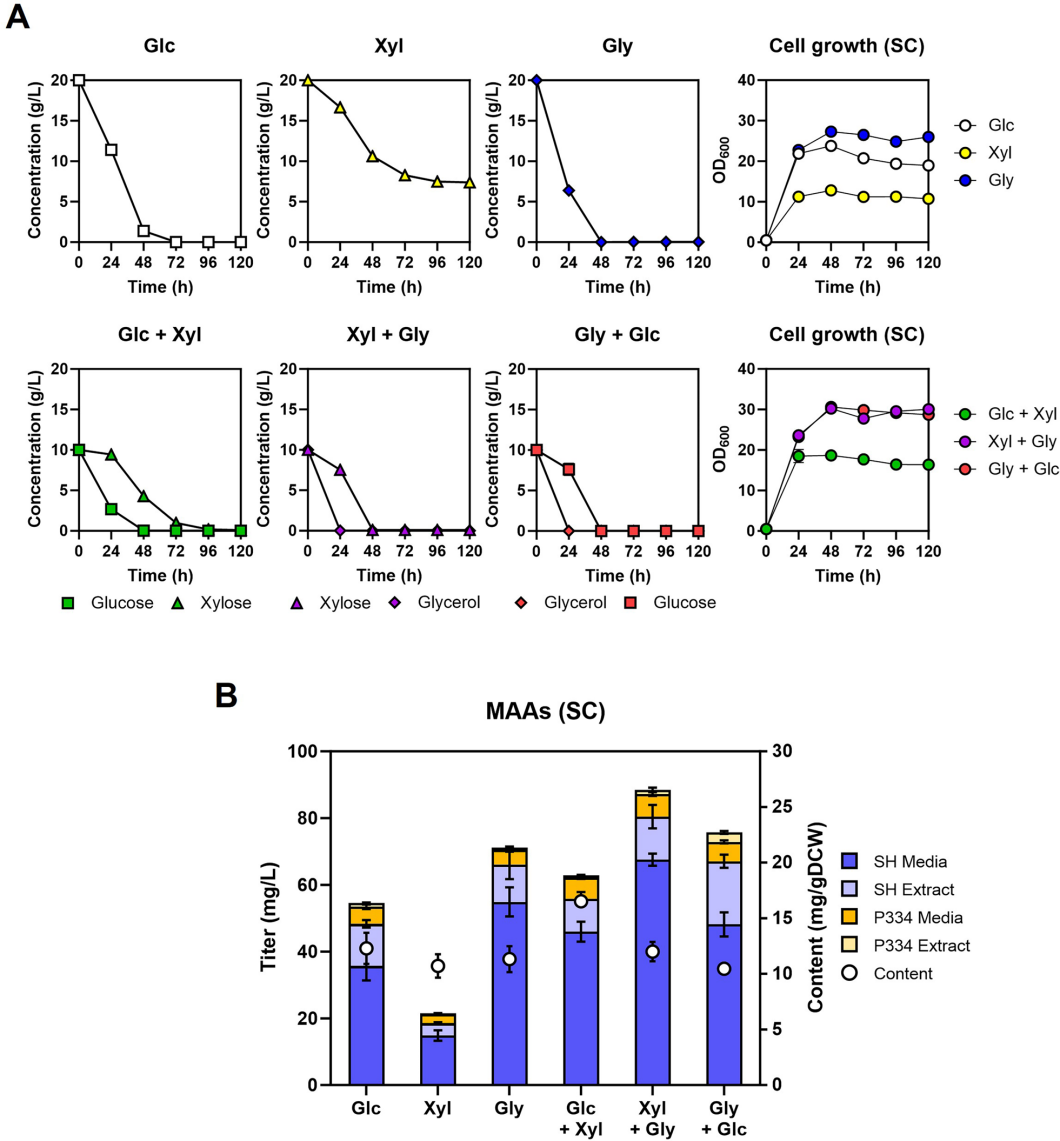
The impact of different carbon sources on MAAs production in SC media. **A** The JHYG831 strain was grown in SC media containing the indicated carbon soruce(s) of 20 g/L in total. Carbon source consumption and optical density (OD) were measured every 24 h. **B** MAAs production levels after 120 h of cultivation. Each value represents the mean ± standard deviation of triplicate samples. *Glc* glucose; *Xyl* xylose; *Gly* glycerol

Using this xylose-utilizing strain JHYG831, we investigated several carbon sources including glucose, glycerol, and xylose for MAAs production. The cells were cultured in SC media containing either a single carbon source or a combination of two carbon sources, with a total concentration of 20 g/L (Fig. 5A). Of the three carbon sources tested, glycerol was found to be the most favorable, followed by glucose and xylose. JHYG831, when grown in glycerol, exhibited the fastest carbon uptake rate at 0.57 g/L·h, in contrast to 0.36 g/L·h in glucose and 0.14 g/L·h in xylose during the initial 24 h. Additionally, the glycerol culture resulted in the highest levels of biomass and MAAs production (Fig. 5A, B) [28]. However, the xylose-utilization pathway in the JHYG831 strain appeared to be inadequate for the complete consumption of all the xylose in the medium (Fig. 5A). Even in the cultures containing two carbon sources, the utilization rate of carbon sources was highest for glycerol, followed by glucose and xylose (Fig. 5A). Despite xylose being an inefficient carbon source, the addition of xylose led to an increase in MAAs production, both in the presence of glucose and glycerol, thereby confirming the role of xylose in supplying S7P through the pentose phosphate pathway (Fig. 5B). As a result, JHYG831 grown in 10 g/L glycerol and 10 g/L xylose produced the highest level of MAAs (88.5 mg/L).

**Fig. 6:**
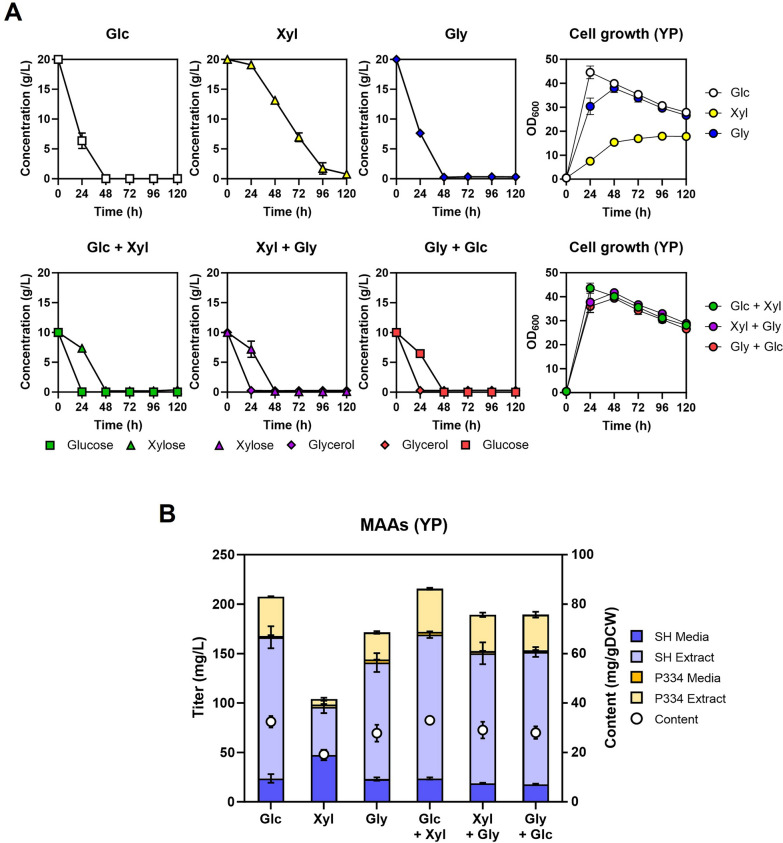
The impact of different carbon sources on MAAs production in YP media. The JHYG831 strain was grown in SC media containing the indicated carbon soruce(s) of 20 g/L in total, and the cell growth and carbon source consumption (**A**) and MAAs production level after 120 h (**B**) were detected. Each value represents the mean ± standard deviation of triplicate samples

We conducted additional tests to examine the impact of various carbon sources on MAAs production in nutrient-rich YP media (Fig. 6A, B). The results showed that the production of MAAs increased by 2 ~ 5 fold in YP media when compared to the production levels in SC media for all tested carbon sources (Fig. 6B). The beneficial impact of xylose was also evident in YP media when used alongside glucose or glycerol as a co-substrate (Fig. 6B). However, in contrast to the pattern observed in SC media, the highest level of MAAs production (215.5 mg/L) was observed in YP medium containing 10 g/L glucose and 10 g/L xylose. Although glycerol was the most preferred carbon source both in SC (Fig. 5A) and YP media (Fig. 6A), the impact of glycerol on cell biomass and MAAs production was less significant in YP media (Fig. 6A, B) compared to SC media (Fig. 5A, B). Additionally, when cells were cultured in YP media, MAAs were predominantly found inside the cells (Fig. 6B), whereas in SC media, they were mostly secreted (Fig. 5B). These results indicate that there are distinct carbon source-dependent metabolic fluxes and expression of potential transporters for MAAs in SC and YP media. The biosynthetic gene clusters in cyanobacteria often contain genes that encode transporters [29, 30]. Nevertheless, no homologous genes were found in the *Y. lipolytica* genome, potentially attributable to the different transporter architecture between prokaryotes and eukaryotes. It is plausible that certain efflux pumps play a role in MAAs export. To gain a better understanding of the medium-dependent alterations in the cellular localization of MAAs, further investigations are necessary to unveil the efflux pumps responsible for MAAs transport in *Y. lipolytica*.

### Enhancing MAAs production by increasing the carbon flux towards the pentose phosphate pathway

**Fig. 7:**
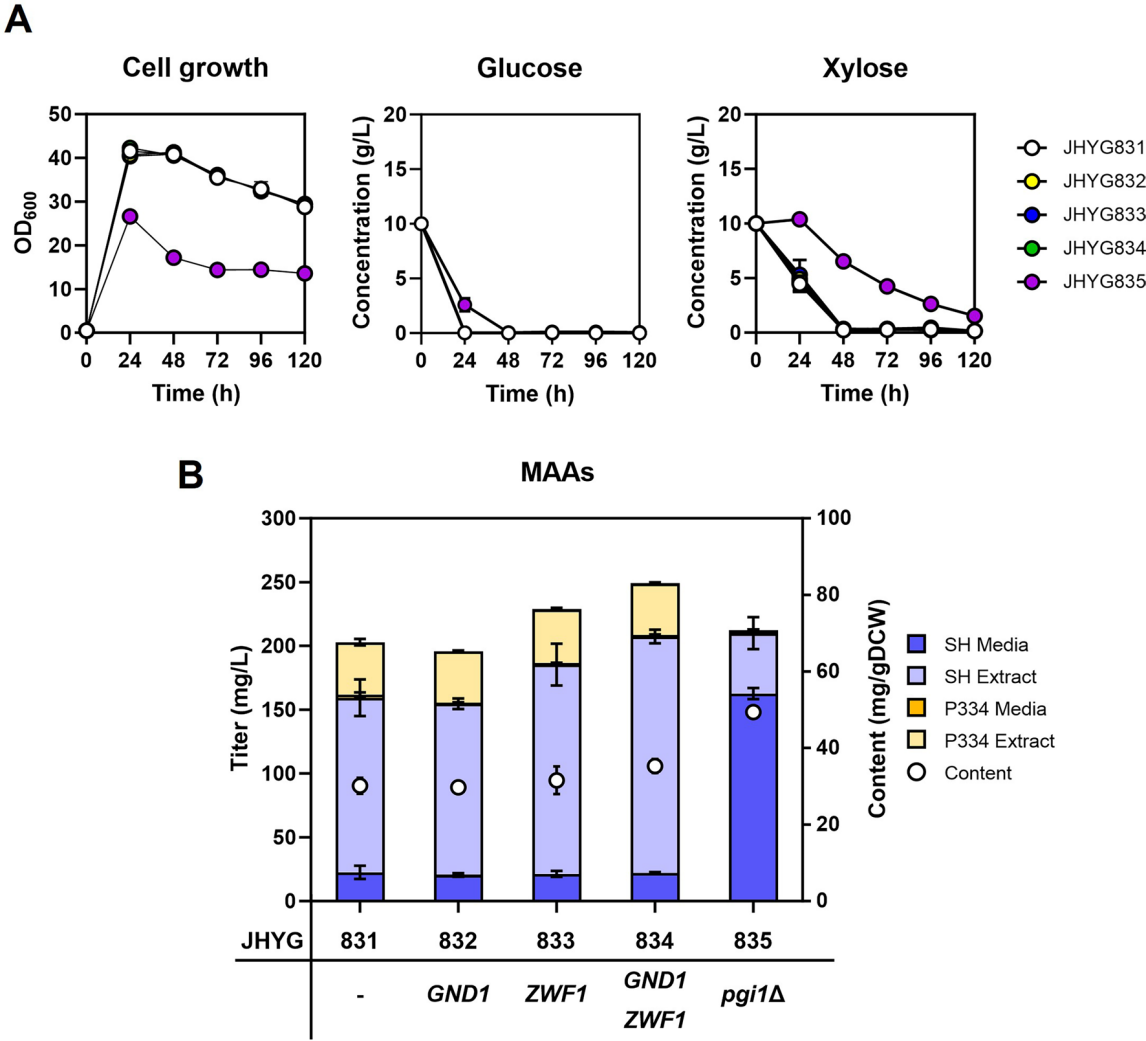
Enhancement of MAAs production via increasing the carbon flux towards the pentose phosphate pathway. The indicated strains were grown in YP media containing 10 g/L glucose and 10 g/L xylose, and the cell growth and carbon source consumption (**A**) and MAAs production level after 120 h (**B**) were detected. Each value represents the mean ± standard deviation of triplicate samples

To increase the production of MAAs even further, we utilized strategies aimed at enhancing the S7P pool. One of the most effective methods was deleting *TAL1*, which encodes a transaldolase that functions in the pentose phosphate pathway. This deletion led to a significant increase in MAAs production in *S. cerevisiae* [15]. However, despite our efforts, we were unable to delete the *TAL1* gene in *Y. lipolytica*, suggesting that *TAL1* may be an essential gene in this organism. Therefore, to enhance the carbon flux towards pentose phosphate pathway, we overexpressed the *ZWF1* and *GND1* genes encoding glucose 6-phosphate dehydrogenase and 6-phosphogluconate dehydrogenase, respectively (Fig. 1A). Overexpression of *ZWF1* alone or together with *GND1* increased MAAs production levels (Fig. 7A, B). JHYG834 overexpressing both *ZWF1* and *GND1* produced the highest level of MAAs (207.4 mg/L of shinorine and 41.6 mg/L of porphyra-334) in YP medium containing 10 g/L glucose and 10 g/L xylose.

We also tried to divert the carbon flux from the glycolysis to the pentose phosphate pathway by deleting the *PGI1* encoding glucose 6-phosphate isomerase, the second enzyme in the glycolysis that converts glucose 6-phosphate (G6P) to fructose 6-phosphate (F6P) (Fig. 1A). The *PGI1* deletion mutant JHYG835 showed a defect in cell growth and carbon source uptake (Fig. 7A) but showed similar MAAs production levels to those of the parental strain JHYG831 (Fig. 7B). Therefore, even with the growth defect caused by *PGI1* deletion, the enhanced carbon flux towards the pentose phosphate pathway may contribute to MAAs production through providing more S7P. Interestingly, MAAs produced in JHYG835 strain were mostly secreted even in YP medium.

## Discussion

MAAs have gained significant attention as natural sunscreens, but the low efficiency of extracting MAAs from natural sources, such as red algae, has led to the exploration of heterologous hosts for MAAs production. Various prokaryotes, including *Escherichia coli*, cyanobacteria, *Corynebacterium glutamicum*, and *Streptomyces* species, and the yeast *S. cerevisiae*, have been employed for MAAs production [10–13, 15]. Notably, *S. cerevisiae* has shown promise as a host to produced MAAs compared to bacterial hosts [14–16]. In this study, we investigated the potential of *Y. lipolytica* as a chassis strain for MAAs production, leveraging its advantages such as a high pentose phosphate pathway flux and the ability to utilize a wide range of carbon sources.

We successfully produced MAAs, mainly shinorine, in *Y. lipolytica* by introducing biosynthetic genes from cyanobacteria and increasing the expression levels of these genes by strengthening the promoter and increasing the gene copy numbers. We also introduced the xylose utilization pathway and investigated the effects of different carbon sources in MAAs production. The addition of xylose as a co-substrate resulted in a significant increase in the production of MAAs in *S. cerevisiae* [15]. Similarly, in *Y. lipolytica*, the utilization of xylose in combination with glucose or glycerol enhanced MAAs production, albeit to a lesser extent compared to *S. cerevisiae*. This observation could be attributed to the naturally higher pentose phosphate pathway flux in *Y. lipolytica*, which results in an increased supply of S7P even without the metabolism of xylose [31, 32]. However, even in *Y. lipolytica*, enhancing the pentose phosphate pathway by overexpressing *ZWF1* and *GND1* genes proved to be effective in further enhancing the production of MAAs.

In addition to the impact of xylose, we observed several distinctions in the production of MAAs between *S. cerevisiae* and *Y. lipolytica*. Firstly, despite we successfully expressed various MysD enzymes (D-Ala-D-Ala ligase homologues) in *S. cerevisiae* [14–16], we were unable to detect shinorine production upon the introduction of *Av.mysE* (NRPS-like enzyme) in *S. cerevisiae* (data not shown). Conversely, both *Np.mysD* and *Av.mysE* were functionally expressed in *Y. lipolytica*, indicating that *Y. lipolytica* is a more suitable chassis strain for expressing *mysE*. Based on the overall production levels of MAAs, including shinorine and porphyra-334, we selected *Np*.MysD over *Av*.MysE for the strain development in this study. However, considering the high specificity of *Av*.MysE towards serine, it would be a more suitable choice when aiming for selective shinorine production.

Secondly, we observed significant differences in the levels of MAAs production and their cellular localization between cultures grown in minimal SC medium and nutrient-rich YP medium. MAAs production levels were more than two-fold higher in YP medium than SC medium. In SC medium, the majority of the MAAs produced was detected in the extracellular environment, whereas in YP medium, a larger proportion of the MAAs was localized within the cells. Interestingly, *PGI1*-deleted *Y. lipolytica* secreted most of the MAAs produced even in YP medium. These findings indicate that the expression of potential transporters responsible for MAAs may be influenced by the type of media used and the cellular carbon metabolism in *Y. lipolytica*. In contrast, when MAAs were produced in *S. cerevisiae*, approximately half of the total amount of MAAs were released into the surrounding medium, and the levels of production and the patterns of secretion demonstrated similarities when SC and YP media were used (Additional file 1: Fig. S1).

While the level of MAAs produced in *Y. lipolytica* is currently lower than that attained in *S. cerevisiae* [14], there is a room for increased enhancement through additional metabolic engineering and optimization of fermentation conditions. Moreover, the distinctive cellular localization of MAAs and the utilization of a wide range of carbon sources could potentially offer advantages of using *Y. lipolytica* for the industrial-scale production of MAAs.

## Conclusions

*Y. lipolytica* was engineered to produce MAAs, predominantly shinorine, by introducing MAA biosynthetic genes from cyanobacteria *N. punctiforme* and *A. variabilis*. Both D-Ala-D-Ala ligase homologue (*Np*.MysD) and NRPS-like enzyme (*Av*.MysE) exhibited functional activity in conjugating amino acids to MG. While *Av*.MysE exclusively produced shinorine, *Np*.MysD showed mixed production of shinorine along with a small amount of porphyra-334. The production of MAAs showed variability based on carbon sources and media types, yet co-utilizing xylose with glucose or glycerol enhanced MAAs synthesis. By incorporating three copies of the MAA biosynthetic genes and overexpressing *GND1* and *ZWF1*, the engineered strain achieved a MAAs production level of 249.0 mg/L (207.4 mg/L shinorine and 41.6 mg/L porphyra-334) in YP medium containing 10 g/L glucose and 10 g/L xylose. These results highlight the potential of *Y. lipolytica* as a promising candidate for MAAs production. This is particularly attributed to its effective expression of the MysE enzyme, which remains non-functional in *S. cerevisiae*, and its versatile utilization of carbon sources like glycerol.

## Methods

### Strains and culture conditions

*Y. lipolytica* strains used in this study are listed in Table 1. Po1g *ku70Δ* strain was used as a parental strain of all the engineered strains. Yeast cell were cultured in synthetic complete (SC) medium (6.7 g/L yeast nitrogen base without amino acids, and 2 g/L amino acids mixture) or YP medium (10 g/L yeast extract and 20 g/L bacto-peptone) containing 20 g/L carbon source (glucose, xylose, or glycerol). For the first inoculation, yeast cells were cultured in 5 mL medium in 50 mL flask for 24 h. Pre-cultured cells were inoculated to OD_600_ of 0.5 in 10 mL medium in 100 mL flask, and then cultured at 30 °C with shaking at 170 rpm for 120 h.

**Table 1:** Strains used in this study

Strain	Genotype
JHYG0	Po1g *ku70*Δ *ura3*Δ
JHYG1	JHYG0 C3:: loxP-*URA3*-loxP
JHYG2	JHYG0 C3:: P_*TEF(406)*_-*Av.mysE*-T_*CYC1*_-P_*TEF(406)*_-*Av.mysC*-T_*CYC1*_-P_*TEF(406)*_-*Av.mysB*-T_*CYC1*_-P_*TEF(406)*_-*Av.mysA*-T_*CYC1*_-loxP-*URA3*-loxP
JHYG3	JHYG0 C3:: P_*TEF(406)*_-*Np.mysD*-T_*CYC1*_-P_*TEF(406)*_-*Np.mysC*-T_*CYC1*_-P_*TEF(406)*_-*Np.mysB*-T_*CYC1*_-P_*TEF(406)*_-*Np.mysA*-T_*CYC1*_-loxP-*URA3*-loxP
JHYG4	JHYG0 C3:: P_*TEF(406)*_-*Np.mysD*-T_*CYC1*_-P_*TEF(406)*_-*Av.mysC*-T_*CYC1*_-P_*TEF(406)*_-*Av.mysB*-T_*CYC1*_-P_*TEF(406)*_-*Av.mysA*-T_*CYC1*_-loxP-*URA3*-loxP
JHYG5	JHYG0 C3:: P_*TEF(406)*_-*Av.mysE*-T_*CYC1*_-P_*TEF(406)*_-*Np.mysC*-T_*CYC1*_-P_*TEF(406)*_-*Av.mysB*-T_*CYC1*_-P_*TEF(406)*_-*Av.mysA*-T_*CYC1*_-loxP-*URA3*-loxP
JHYG6	JHYG0 C3:: P_*TEF(406)*_-*Av.mysE*-T_*CYC1*_-P_*TEF(406)*_-*Av.mysC*-T_*CYC1*_-P_*TEF(406)*_-*Np.mysB*-T_*CYC1*_-P_*TEF(406)*_-*Av.mysA*-T_*CYC1*_-loxP-*URA3*-loxP
JHYG7	JHYG0 C3:: P_*TEF(406)*_-*Av.mysE*-T_*CYC1*_-P_*TEF(406)*_-*Av.mysC*-T_*CYC1*_-P_*TEF(406)*_-*Av.mysB*-T_*CYC1*_-P_*TEF(406)*_-*Np.mysA*-T_*CYC1*_-loxP-*URA3*-loxP
JHYG8	JHYG0 C3:: P_*UAS1B8-TEF(136)*_-*Np.mysD*-T_*CYC1*_-P_*UAS1B8-TEF(136)*_-*Av.mysC*-T_*CYC1*_-P_*UAS1B8-TEF(136)*_-*Av.mysB*-T_*CYC1*_-P_*UAS1B8-TEF(136)*_-*Av.mysA*-T_*CYC1*_-loxP-*URA3*-loxP
JHYG9	JHYG0 C3:: P_*TEFin*_-*Np.mysD*-T_*CYC1*_-P_*TEFin*_-*Av.mysC*-T_*CYC1*_-P_*TEFin*_-*Av.mysB*-T_*CYC1*_-P_*TEFin*_-*Av.mysA*-T_*CYC1*_-loxP-*URA3*-loxP
JHYG82	JHYG8 E3:: P_*UAS1B8-TEF(136)*_-*Np.mysD*-T_*CYC1*_-P_*UAS1B8-TEF(136)*_-*Av.mysC*-T_*CYC1*_-P_*UAS1B8-TEF(136)*_-*Av.mysB*-T_*CYC1*_-P_*UAS1B8-TEF(136)*_-*Av.mysA*-T_*CYC1*_-loxP-*URA3*-loxP
JHYG83	JHYG82 D1:: P_*UAS1B8-TEF(136)*_-*Np.mysD*-T_*CYC1*_-P_*UAS1B8-TEF(136)*_-*Av.mysC*-T_*CYC1*_-P_*UAS1B8-TEF(136)*_-*Av.mysB*-T_*CYC1*_-P_*UAS1B8-TEF(136)*_-*Av.mysA*-T_*CYC1*_-loxP-*URA3*-loxP
JHYG831	JHYG83 AXP1:: P_*TEF(406)*_-*Ps.XYL1*-T_*CYC1*_-P_*TEF(406)*_-*Ps.XYL2*-T_*CYC1*_-P_*TEF(406)*_-*Yl.XYL3*-T_*CYC1*_-loxP-*URA3*-loxP
JHYG832	JHYG831 A1:: P_*UAS1B8-TEF(136)*_-*GND1*-T_*CYC1*_-lox71-*URA3*-lox66
JHYG833	JHYG831 A1:: P_*UAS1B8-TEF(136)*_-*ZWF1*-T_*CYC1*_-lox71-*URA3*-lox66
JHYG834	JHYG831 A1:: P_*UAS1B8-TEF(136)*_-*GND1*-T_*CYC1*_-P_*UAS1B8-TEF(136)*_-*ZWF1*-T_*CYC1*_-lox71-*URA3*-lox66
JHYG835	JHYG831 *pgi1*Δ:: lox71-*URA3*-lox66
JHYGX1	JHYG0 AXP1:: P_*TEF(406)*_-*Yl.XYL1*-T_*CYC1*_-P_*TEF(406)*_-*Yl.XYL2*-T_*CYC1*_-P_*TEF(406)*_-*Yl.XYL3*-T_*CYC1*_-loxP-*URA3*-loxP
JHYGX2	JHYG0 AXP1:: P_*TEF(406)*_-*Ps.XYL1*-T_*CYC1*_-P_*TEF(406)*_-*Ps.XYL2*-T_*CYC1*_-P_*TEF(406)*_-*Yl.XYL3*-T_*CYC1*_-loxP-*URA3*-loxP

**Table 2:** Plasmids used in this study

Plasmid	Description
pYL-Cre-LEU2	Cre recombinase expression plasmid containing P_*EXP1*_-Cre-T_*CYC1*_ and *LEU2* marker
Int C3 TEF AAAA	C3 site integration plasmid, P_*TEF(406)*_-*Av.mysE*-T_*CYC1*_-P_*TEF(406)*_-*Av.mysC*-T_*CYC1*_-P_*TEF(406)*_-*Av.mysB*-T_*CYC1*_-P_*TEF(406)*_-*Av.mysA*-T_*CYC1*_-loxP-*URA3*-loxP
Int C3 TEF NNNN	C3 site integration plasmid, P_*TEF(406)*_-*Np.mysD*-T_*CYC1*_-P_*TEF(406)*_-*Np.mysC*-T_*CYC1*_-P_*TEF(406)*_-*Np.mysB*-T_*CYC1*_-P_*TEF(406)*_-*Np.mysA*-T_*CYC1*_-loxP-*URA3*-loxP
Int C3 TEF AAAN	C3 site integration plasmid, P_*TEF(406)*_-*Np.mysD*-T_*CYC1*_-P_*TEF(406)*_-*Av.mysC*-T_*CYC1*_-P_*TEF(406)*_-*Av.mysB*-T_*CYC1*_-P_*TEF(406)*_-*Av.mysA*-T_*CYC1*_-loxP-*URA3*-loxP
Int C3 TEF AANA	C3 site integration plasmid, P_*TEF(406)*_-*Av.mysE*-T_*CYC1*_-P_*TEF(406)*_-*Np.mysC*-T_*CYC1*_-P_*TEF(406)*_-*Av.mysB*-T_*CYC1*_-P_*TEF(406)*_-*Av.mysA*-T_*CYC1*_-loxP-*URA3*-loxP
Int C3 TEF ANAA	C3 site integration plasmid, P_*TEF(406)*_-*Av.mysE*-T_*CYC1*_-P_*TEF(406)*_-*Av.mysC*-T_*CYC1*_-P_*TEF(406)*_-*Np.mysB*-T_*CYC1*_-P_*TEF(406)*_-*Av.mysA*-T_*CYC1*_-loxP-*URA3*-loxP
Int C3 TEF NAAA	C3 site integration plasmid, P_*TEF(406)*_-*Av.mysE*-T_*CYC1*_-P_*TEF(406)*_-*Av.mysC*-T_*CYC1*_-P_*TEF(406)*_-*Av.mysB*-T_*CYC1*_-P_*TEF(406)*_-*Np.mysA*-T_*CYC1*_-loxP-*URA3*-loxP
Int C3 UAS1B8 AAAN	C3 site integration plasmid, P_*UAS1B8-TEF(136)*_-*Np.mysD*-T_*CYC1*_-P_*UAS1B8-TEF(136)*_-*Av.mysC*-T_*CYC1*_-P_*UAS1B8-TEF(136)*_-*Av.mysB*-T_*CYC1*_-P_*UAS1B8-TEF(136)*_-*Av.mysA*-T_*CYC1*_-loxP-*URA3*-loxP
Int C3 TEFin AAAN	C3 site integration plasmid, P_*TEFin*_-*Np.mysD*-T_*CYC1*_-P_*TEFin*_-*Av.mysC*-T_*CYC1*_-P_*TEFin*_-*Av.mysB*-T_*CYC1*_-P_*TEFin*_-*Av.mysA*-T_*CYC1*_-loxP-*URA3*-loxP
Int E3 UAS1B8 AAAN	E3 site integration plasmid, P_*UAS1B8-TEF(136)*_-*Np.mysD*-T_*CYC1*_-P_*UAS1B8-TEF(136)*_-*Av.mysC*-T_*CYC1*_-P_*UAS1B8-TEF(136)*_-*Av.mysB*-T_*CYC1*_-P_*UAS1B8-TEF(136)*_-*Av.mysA*-T_*CYC1*_-loxP-*URA3*-loxP
Int D1 UAS1B8 AAAN	D1 site integration plasmid, P_*UAS1B8-TEF(136)*_-*Np.mysD*-T_*CYC1*_-P_*UAS1B8-TEF(136)*_-*Av.mysC*-T_*CYC1*_-P_*UAS1B8-TEF(136)*_-*Av.mysB*-T_*CYC1*_-P_*UAS1B8-TEF(136)*_-*Av.mysA*-T_*CYC1*_-loxP-*URA3*-loxP
Int AXP1 TEF Yl.XYL	AXP1 site integration plasmid, P_*TEF(406)*_-*Yl.XYL1*-T_*CYC1*_-P_*TEF(406)*_-*Yl.XYL2*-T_*CYC1*_-P_*TEF(406)*_-*Yl.XYL3*-T_*CYC1*_-loxP-*URA3*-loxP
Int AXP1 TEF XYL	AXP1 site integration plasmid, P_*TEF(406)*_-*Ps.XYL1*-T_*CYC1*_-P_*TEF(406)*_-*Ps.XYL2*-T_*CYC1*_-P_*TEF(406)*_-*Yl.XYL3*-T_*CYC1*_-loxP-*URA3*-loxP
Int A1 UAS1B8 GND1	A1 site integration plasmid, P_*UAS1B8-TEF(136)*_-*GND1*-T_*CYC1*_-lox71-*URA3*-lox66
Int A1 UAS1B8 ZWF1	A1 site integration plasmid, P_*UAS1B8-TEF(136)*_-*ZWF1*-T_*CYC1*_-lox71-*URA3*-lox66
Int A1 UAS1B8 GND1-ZWF1	A1 site integration plasmid, P_*UAS1B8-TEF(136)*_-*GND1*-T_*CYC1*_-P_*UAS1B8-TEF(136)*_-*ZWF1*-T_*CYC1*_-lox71-*URA3*-lox66
Del PGI1 Δ	*PGI1* deletion plasmid, *PGI1* up 1 kb-lox71-*URA3*-lox66-*PGI1* down 1 kb

### Construction of plasmids and strains

Plasmids used in this study are listed in Table 2. *Y. lipolytica* codon optimized *Ava3858* (*mysA*), *Ava3857* (*mysB*), *Ava3856* (*mysC*), and *Ava3855* (*mysD*) genes from *A. variabilis* and *NpR5600* (*mysA*), *NpR5599* (*mysB*), *NpR5598* (*mysC*), and *NpF5597* (*mysE*) genes from *N. punctiforme* were synthesized. Xylose assimilation genes *Ps.XYL1* and *Ps.XYL2* from *P. stipitis* were amplified from coex416-XYL [15]. Plasmids were generated by standard molecular cloning methods with restriction enzymes and DNA ligases. Plasmids for gene integration contain gene expression cassette(s) controlled by P_*UAS1B8-TEF(136)*_-T_*CYC1*_ or P_*TEF(406)*_-T_*CYC1*_ and loxP (or lox71)-*URA3*-loxP (or lox66) [33], which are flanked by 1-kb upstream and downstream regions of the integration sites, A1, C3, D1, or E3 [34]. The Del PGI1Δ plasmid contains lox71-*URA3*-lox66 flanked by 1-kb upstream and downstream regions of the *PGI1* ORF. The integration or deletion plasmids were linearized by cutting with *Nde*I or *Smi*I, and transformed into *Y. lipolytica* Po1g. The transformants were selected in SC medium lacking uracil. The *URA3* marker recovery for additional gene introduction was performed through the following steps. A pYL-Cre-LEU2 plasmid, containing *LEU2* marker and the Cre recombinase gene under the control of the *EXP1* promoter, was transformed into *Y. lipolytica* and selected on SC-Leu medium. Subsequently, the colonies were cultured in 5 mL of YPD media for 24 h at 30 °C to promote plasmid loss, then plated on YPD. The clones with LEU^−^ and URA^−^ phenotype were selected by patching the colonies on SC-Leu and SC-Ura media.

### Analytical methods

Cell growth was determined by measuring optical density at 600 nm with spectrophotometer (Varian Cary 50 UV − vis). To detect the amount of remained carbon sources, 500 μL of cell supernatant was filtered with 0.22 μm PVDF syringe filter, and then analyzed by HPLC. UltiMate 3000 HPLC system (Thermo Fisher Scientific) equipped with an Aminex HPX-87H column (300 mm × 7.8 mm, 5 μm, Bio-Rad) and a refractive index (RI) detector. Mobile phase is water with 0.05% (v/v) H_2_SO_4_ at 0.6 mL/min flow rate and 60 °C.

To prepare MAAs in the medium, the cell supernatants were filtered using a 0.22 μm PVDF syringe filter. For MAAs in the cell, 100–500 μL cell broth was collected, resuspended in 500 μL of water, and mixed with 750 μL chloroform. The cells were disrupted by vortexing for 10 min. The upper water layer obtained after centrifugation was filtered using a 0.22 μm PVDF syringe filter, and then analyzed by UltiMate 3000 HPLC system (Thermo Fisher Scientific) equipped with an Agilent Eclipse XDB-C18 column (5 μm, 4.6 × 250 mm) and a UV–vis detector. A UV–vis detector was used at 334 nm. The mobile phase used was water: acetonitrile = (95: 5, v/v) with 0.1% (v/v) TFA at 0.5 mL/min flow rate and 40 °C. Dry cell weight (DCW) was measured after drying 1 mL cell pellet at 60 °C oven.

### Supplementary Information

**Additional file 1: Figure S1.** Production of MAAs in *S. cerevisiae* in SC and YP media*.*

## Data Availability

Data availability statement is not applicable.
