# Supplementary material for: Efficient production of mycosporine-like amino acids, natural sunscreens, in Yarrowia lipolytica
Source: Biotechnol Biofuels Bioprod. 2023 Oct 29;16:162. doi: 10.1186/s13068-023-02415-y (PMC10614408; doi:10.1186/s13068-023-02415-y)
Supplement: Supplementary file 1 — Additional file 1: Figure S1. Production of MAAs in S. cerevisiae in SC and YP media. [file 13068_2023_2415_MOESM1_ESM.docx]

**Additional file 1**

**Fig. S1** Production of MAAs in *S. cerevisiae* in SC and YP media*.* The JHSM132 strain [14] was grown SC or YP media containing 10 g/L glucose and 10 g/L xylose. After 120 h of cultivation, MAAs production levels in media and cell extracts were measured.
